# Revised and updated nomenclature for highly pathogenic avian influenza A (H5N1) viruses: World Health Organization/World Organisation for Animal Health/Food and Agriculture Organization (WHO/OIE/FAO) H5N1 Evolution Working Group*

**DOI:** 10.1111/irv.12230

**Published:** 2014-01-31

**Keywords:** H5N1, hemagglutinin, highly pathogenic avian influenza, molecular epidemiology, nomenclature, phylogenetics, viral evolution

## Abstract

The divergence of the hemagglutinin gene of A/goose/Guangdong/1/1996-lineage H5N1 viruses during 2011 and 2012 (807 new sequences collected through December 31, 2012) was analyzed by phylogenetic and p-distance methods to define new clades using the pre-established nomenclature system. Eight new clade designations were recommended based on division of clade 1·1 (Mekong River Delta), 2·1·3·2 (Indonesia), 2·2·2 (India/Bangladesh), 2·2·1·1 (Egypt/Israel), and 2·3·2·1 (Asia). A simplification to the previously defined criteria, which adds a letter rather than number to the right-most digit of fifth-order clades, was proposed to facilitate this and future updates.

## Introduction

The need for a system to classify divergent groups of the A/goose/Guangdong/1/1996 lineage of Eurasian highly pathogenic avian influenza (HPAI) A(H5N1) viruses was first recognized in 2008.^1^ The WHO/OIE/FAO H5N1 Evolution Working Group developed criteria used to distinguish variant groups of the H5 hemagglutinin (HA) gene and a nomenclature system to define clades.^1^–^3^ Using phylogenetic analyses and nucleotide sequence divergence calculations, H5N1 virus clades were based on sharing of a common ancestral node and monophyletic evolution with a bootstrap value of ≥60 at the clade-defining node (after at least 1000 bootstrap replicates and/or significant Bayesian posterior probabilities). Individual groups also maintained an average within-clade percentage pairwise nucleotide distance of ≤1·5%.^1^ The continuous evolution of the H5 HA since the last nomenclature update (data collection through January 2011) has resulted in additional phylogenetic groups that exceeded the clade boundaries designated previously.^3^ Therefore, the WHO/OIE/FAO H5N1 Evolution Working Group has examined the HA sequence data available as of December 31, 2012 to update this classification system.

## Materials and methods

### Hemagglutinin gene data sets

A total of 7729 hemagglutinin nucleotide sequences were obtained from GISAID and GenBank. Sequences had submission dates up to and including December 31, 2012. Only H5N1 subtype viruses were considered, with genetically manipulated strains excluded from the analysis. Data were aligned via MAFFT v7.015b 4 and trimmed to the beginning of the mature H5 HA protein gene sequence using JalView v2.8.^5^ Redundant sequences were removed when identical strain names were identified. Sequences that caused alignment frameshifts without corresponding nucleotide insertions, which were <60% of the trimmed alignment length and with more than 5 ambiguous nucleotides were removed. Maximum likelihood trees (GTR+GAMMA with 10 000 local support bootstraps) were constructed from the remaining 3713 HA sequences using FastTree v2.1.4.^6^ Data S2 contain a listing of all sequences used for clade analysis in this study, their assigned clades, accession numbers, and data sources. Data S3 contain a list of authors, originating and submitting laboratories of the sequences from GISAID.

### Clade annotation/p-distance comparisons

**Figure 1:**
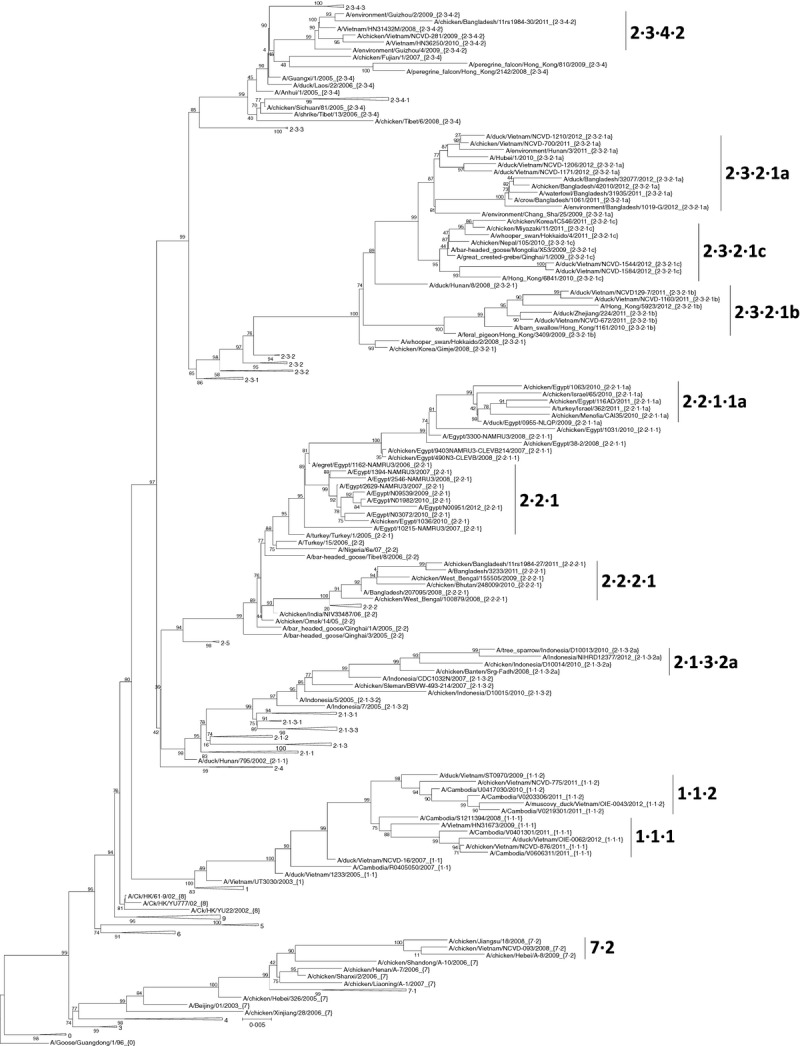
Phylogenetic relationships of recently diverged A/goose/Guangdong /1/1996-like H5 hemagglutinin (HA) genes. A maximum likelihood tree of 198 HA nucleotide sequences from H5N1 viruses was constructed with 10 000 local support bootstraps (above branches) using FastTree2 (GTR+GAMMA) and rooted to A/goose/Guangdong /1/1996. Newly designated clades are underlined. Solid triangles denote HA clades of viruses that have not been in circulation since 2010 or earlier. Scale bar denotes nucleotide substitutions per site. Sequence data (FASTA) used to generate this figure are provided in Data S4.

Previously unclassified sequences (*n* = 766) were annotated by clade using LABEL v0.4^7^ (http://label.phiresearchlab.org) and confirmed by phylogenetic analysis for accuracy. For the evaluation of newly proposed clade definitions, phylogenetic clustering was performed using FastTree2 with secondary corroboration using ExaML v1.0.0^8^ in conjunction with RaxML v7.4.2.^9^–^11^ Following identification of pre-existing clades across the large phylogenetic trees generated (Figure S1A–E), the average pairwise nucleotide distance (APD) of each clade was calculated using distance matrices computed in MEGA v5.1^12^ (Figure S1; Table 1). The figure PDFs were also generated in MEGA. Newly designated clades were required to include samples collected in 2011 and/or 2012 that formed monophyletic clusters with bootstrap values ≥60% (10 000 local support bootstraps, FastTree2) and within-clade APDs of ≤1·5%. New clades were evaluated in the context of five major phylogenetic groups identified: (i) clades 0, 1, and 3 through 9; (ii) clades 2·1 and 2·4; (iii) clades 2·2 and 2·5; (iv) clade 2·3 with all sublineages except 2·3·2·1; (v) clade 2·3·2·1 (Figure S1A–E). Following identification of new clades, phylogenetic relationships of a representative group of 198 HA genes were performed using a maximum likelihood tree constructed with 10 000 local support bootstraps using FastTree2 (GTR+GAMMA) and rooted to A/goose/Guangdong /1/1996 (Figure1). Sequence data used to generate this figure are provided in Data S4.

**Table 1:** Current and proposed clade designations by average pairwise distances

Previous clade designation	Intraclade Average p-distance, %	New clade	Intraclade Average p-distance, %
1·1	2·1	1·1·1	1·44
		1·1·2	1·52
2·1·3·2	1·9	2·1·3·2a	1·53
2·2·1	1·6	No additional split	
2·2·1·1	1·9	2·2·1·1a	1·28
2·2·2	1·7	2·2·2·1	1·46
2·3·2·1	2·4	2·3·2·1a	1·48
		2·3·2·1b	1·53
		2·3·2·1c	1·21
2·3·4·2	1·6	No additional split	
7·2	2·0	No additional split	

A modification of the previous H5N1 nomenclature system was used for this update to simplify the designation of fifth-order clades. The nomenclature update reported in October 2011 included 8 clades with fourth-order designations requiring four-digit numerals separated by decimals (e.g., clades 2·2·1·1, 2·1·3·2, 2·3·2·1).^3^ With the observation that fifth-order clades would arise from this analysis, a recommendation to define the fifth-order groups using an additional letter, rather than a number to the right-most digit of fourth-order clades, was implemented (i.e., 2·3·2·1a). Although the numerical system has been effective to capture clade ancestry in the past, there was concern that new designations exceeding four digits would impair communication within the scientific community and public.

## Results

Phylogenetic analysis identified clustering of monophyletic groups in the majority of circulating clades. New sequences that had not been previously classified were first grouped according to clade based on the established nomenclature criteria. Groups that contained viruses collected during 2011 and 2012 were analyzed to determine the within-clade average pairwise nucleotide distances (Figure S1A–E). Based on previously defined criteria, we concluded that clades 1·1, 2·1·3·2, 2·2·1·1, 2·2·2, and 2·3·2·1 required splitting (Table 1). Clade 1·1 had an internal average pairwise distance (APD) of 2·1% with viruses detected throughout 2011 and 2012 in Vietnam and Cambodia and was split into clades 1·1·1 and 1·1·2. Clade 2·1·3·2 had an internal APD of 1·9% with continuing evolution through 2011 and 2012 in Indonesia. This group was split into one additional clade, termed 2·1·3·2a, using a letter to signify the fifth-order group. Clade 2·2·1·1 in Egypt and Israel had an internal APD of 1·9% with continuing evolution through 2011 and was subdivided into clade 2·2·1·1a. Clade 2·2·2 had an internal APD of 1·7% and viruses detected in India, Bangladesh, Bhutan, and Nepal during 2010 and 2011. This split resulted in clade 2·2·2·1. Finally, clade 2·3·2·1 had the highest within-clade APD (2·4%) and required splitting into three individual clades (2·3·2·1a-c) due to the emergence of distinct monophyletic groups each with high bootstrap support. Clades 2·2·1, 2·3·4·2, and 7·2 were just above the 1·5% threshold but were not split due to lack of either sufficient circulation in 2011 and 2012 (clade 7·2) and/or formation of new monophyletic groups during this time period (2·2·1 and 2·3·4·2). No changes were identified in clades that were previously considered “extinct” as no new data were identified. However, no new virus sequences were identified in either 2011 or 2012 in several clades including 7·1, 2·1·3·1, 2·1·3·3, 2·2·2, 2·3·4·1, and 2·3·4·3 (Figure1).

## Discussion

H5N1 viruses have become enzootic in several geographically isolated regions of the world with little or no epizootiologic association or gene flow between them. The October 2011 update of the WHO/OIE/FAO H5N1 Evolution Working Group reported twelve H5 clades that circulated in the previous 3 years. To address the question of whether continued evolution has led to the appearance of new clades, we initially constructed a phylogenetic tree composed of more than 3500 HA sequences. As expected, the addition of more than 750 new sequences produced a number of monophyletic groups within the H5N1 clades circulating during 2011 and 2012. After measuring within- and between-group average nucleotide pairwise distances, it was observed that several clades required splitting into one or more newly defined higher-order clades. The current analysis proposes the designation of eight new clades that meet the criteria of the H5N1 nomenclature system. Furthermore, the emergence of five fifth-order clades called for the proposal of a simplified convention that adds a letter, rather than another number, to the right-most digit of fourth-order groups to increase the value. This additional designation will facilitate description of clades that have reached a high level of genetic diversity, while maintaining an alphanumeric connection to ancestral sequences. For the next update of the H5 clade nomenclature, those fifth-order groups that eventually reach a sixth-order would have the next letter in the alphabet added. Assuming no change in the rates of H5 HA evolution and estimating that a new clade would emerge approximately every 1·5–2 years, no additional characters would be needed until ∼2050.

Despite many successful efforts to eliminate or control the spread of H5N1 in poultry, viruses persist in enzootic regions and occasionally seed epizootics in other areas via poultry trade and related activity or through wild birds. As a consequence, the classification of H5N1 viruses based on HA evolution requires periodic updating, making classification dynamic as the virus has expanded within several disparate ecosystems and along distinct evolutionary trajectories. Clade 1 viruses have persisted in poultry populations in the Mekong River Delta since 2003, and their active circulation in the region is evident from the designation of new clades 1·1·1 and 1·1·2.^13^ Clade 2·1 viruses have circulated since 2003 in Indonesia, and their post-2010 evolution resulted in a single new clade termed 2·1·3·2a. Clade 2·2·1·1 viruses were enzootic in Egypt with detection also in Israel through 2011 and evolved into a newly designated clade 2·2·1·1a, which appears to still be maintained primarily within the commercial poultry sector.^14^ Clade 2·2·2 was enzootic in Bangladesh and neighboring countries resulting in clade 2·2·2·1. Clade 2·3·2·1 revealed the greatest divergence, resulting in three newly designated clades. Clade 2·3·2·1a (provisionally designated as A/Hubei/1/2010-like) has been dominant in Vietnam since as early as 2009 but was also detected in Bangladesh and neighboring countries in recent years.^15^ Clade 2·3·2·1b (A/barn-swallow/HK/1161/2010-like) circulated only in China, Hong Kong SAR, and Vietnam. Clade 2·3·2·1c (represented by A/Hong Kong/6841/2010) has circulated broadly in domestic and wild birds and was reported in many Asian countries, but more recently in Indonesia and Vietnam.^16^ The high within-clade APD (2·4%) prior to these new splits and the diversification of clade 2·3·2·1 into three fifth-order clades was likely due to rapid geographic expansion and establishment of enzootic foci in many disparate regions. Finally, as described in previous updates, the expansion of some clades was countered by the lack of detection of other clades since 2010 or before (Figure1). Continued surveillance, monitoring, and characterization of H5N1 avian influenza viruses will be critical to assess the prevalence of these new clades in the years to come.

## Supporting Information

**Figure S1.** (A–E). Phylogenetic relationships of recently diverged A/goose/Guangdong /1/1996-like H5 hemagglutinin (HA) genes.

**Data S1.** Members of the World Health Organization/World Organisation for Animal Health/Food and Agriculture Organization (WHO/OIE/FAO) H5N1 Evolution Working Group.

**Data S2.** List of viruses, clade designation, gene accession numbers, and database sources.

**Data S3.** List of authors, originating and submitting laboratories of the sequences from GISAID's EpiFlu™ Database used in this report.

**Data S4.** H5 nucleotide sequence alignment used for the phylogenetic tree shown in Figure 1.
