# Supplementary material for: Revised and updated nomenclature for highly pathogenic avian influenza A (H5N1) viruses: World Health Organization/World Organisation for Animal Health/Food and Agriculture Organization (WHO/OIE/FAO) H5N1 Evolution Working Group
Source: Influenza Other Respir Viruses. 2014 Jan 31;8(3):384–8. doi: 10.1111/irv.12230 (PMC4181488; doi:10.1111/irv.12230)
Supplement: Supplementary file 6 — Data S1. Members of the World Health Organization/World Organisation for Animal Health/Food and Agriculture Organization (WHO/OIE/FAO) H5N1 Evolution Working Group. [file irv0008-0384-SD6.docx]

**SUPPLEMENTARY DATA S1**

**H5N1 Evolution Working Group Members and Collaborators**

The working group was established in 2008 by request of the World Health Organization’s Global Influenza Programme, Department of Epidemic and Pandemic Alert and Response (WHO, GIP, EPR), the World Organization for Animal Health (OIE), and the Food and Agriculture Organization (FAO). It currently consists of the following persons:

1. Justin Bahl, Duke-NUS Graduate Medical School; Singapore and Centers for Disease Control and Prevention, Atlanta, Georgia, USA;
2. Terry Besselaar, WHO, GISRS, Geneva, Switzerland;
3. Ian H. Brown, Veterinary Laboratories Agency, Addlestone, England, United Kingdom;
4. Ilaria Capua, Istituto Zooprofilattico Sperimentale delle Venezie, Padova, Italy;
5. Giovanni Cattoli, Istituto Zooprofilattico Sperimentale delle Venezie, Padova, Italy;
6. Hualan Chen, Harbin Veterinary Research Institute, CAAS, China;
7. Nancy Cox, WHO Collaborating Centre for the Surveillance, Epidemiology and Control of Influenza, Centers for Disease Control and Prevention, Atlanta, Georgia, USA;
8. Filip Claes, FAO, Rome, Italy;
9. Gwenaelle Dauphin, FAO, AGAH, Rome, Italy;
10. C. Todd Davis, Centers for Disease Control and Prevention, Atlanta, Georgia, USA;
11. Ruben O. Donis, Centers for Disease Control and Prevention, Atlanta, Georgia, USA;
12. Ron A.M. Fouchier, Erasmus University, Netherlands;
13. Yi Guan, The University of Hong Kong, HK SAR, China;
14. Keith Hamilton, OFFLU, OIE, Paris, France;
15. Yunho Jang, Centers for Disease Control and Prevention, Atlanta, Georgia, USA;
16. Yoshihiro Kawaoka, University of Wisconsin, Madison, Wisconsin, USA and Institute of Medical Science, University of Tokyo, Tokyo, Japan;
17. Anne Kelso, WHO Collaborating Centre for Reference and Research on Influenza, Melbourne, Australia;
18. John McCauley, WHO Collaborating Centre for Reference and Research on Influenza,
    MRC National Institute for Medical Research, London, UK;
19. Elizabeth Mumford, WHO, GIP, EPR, Geneva, Switzerland;
20. Teguh Prajitno, Japfa, Jakarta, Indonesia;
21. Colin A. Russell, Department of Zoology, University of Cambridge, England, United Kingdom;
22. Derek Smith, Department of Zoology, University of Cambridge, England, United Kingdom;
23. Gavin J.D. Smith, Duke-NUS Graduate Medical School; Singapore;
24. Yuelong Shu, Chinese Center for Disease Control and Prevention, Beijing, China;
25. Masato Tashiro, WHO Collaborating Centre for Reference and Research on Influenza,
    National Institute of Infectious Diseases, Tokyo, Japan;
26. Samuel Shepard, Centers for Disease Control and Prevention, Atlanta, Georgia, USA;
27. David Suarez, USDA, ARS, Athens, USA;
28. Dhanasekaran Vijaykrishna, Duke-NUS Graduate Medical School; Singapore;
29. Richard Webby, WHO Collaborating Center for Studies on the Ecology of Influenza in Animals, St. Jude Children’s Research Hospital, Memphis, USA;
30. Robert Webster, St. Jude Children's Research Hospital, Memphis, USA;
31. Frank Wong, Australian Animal Health Laboratory, Geelong, Australia;
